# A multi-method approach to selecting PRO-CTCAE symptoms for patient-reported outcome in women with endometrial or ovarian cancer undergoing chemotherapy

**DOI:** 10.1186/s41687-023-00611-w

**Published:** 2023-07-18

**Authors:** Mille Guldager Christiansen, Helle Pappot, Pernille Tine Jensen, Mansoor Raza Mirza, Mary Jarden, Karin Piil

**Affiliations:** 1 Department of Oncology, Centre for Cancer and Organ Diseases, Copenhagen University Hospital, Rigshospitalet, Copenhagen, Denmark; 2 Faculty of Health and Medical Sciences, University of Copenhagen, Copenhagen, Denmark; 3 Department of Clinical Medicine, Faculty of Health and Medical Sciences, University of Copenhagen, Copenhagen, Denmark; 4 Department of Gynecology and Obstetrics, Aarhus University Hospital, Aarhus, Denmark; 5 Department of Clinical Medicine, Faculty of Health, University of Aarhus, Aarhus, Denmark; 6 Department of Haematology, Centre for Cancer and Organ Diseases, Copenhagen University Hospital, Rigshospitalet, Copenhagen, Denmark; 7 Department of People and Technology, Roskilde University, Roskilde, Denmark

**Keywords:** Item selection, Patient-reported outcomes, PRO, Ovarian cancer, Endometrial cancer, PRO-CTCAE library, Chemotherapy

## Abstract

**Background:**

Women with endometrial or ovarian cancer experience a variety of symptoms during chemotherapy. Patient-Reported outcomes (PROs) can provide insight into the symptoms they experience. A PRO tool tailored to this patient population can help accurately monitor adverse events and manage symptoms. The objective of this study was to identify items in the National Cancer Institute’s measurement system Patient-Reported Outcomes Version of the Common Terminology Criteria for Adverse Events (PRO-CTCAE®) appropriate for use in a PRO tool for a population of women with endometrial or ovarian cancer undergoing treatment with taxanes (paclitaxel or docetaxel) in combination with carboplatin.

**Methods:**

A two-phase, sequential multi-methods approach was applied. In phase one, a comprehensive literature search was done to map the toxicity of the applied chemotherapeutics and phase III clinical studies. Phase two, which comprised selecting the PRO-CTCAE items, included discussions with and feedback from a patient advisory board, an additional literature search, and focus group interviews with senior oncologists and specialized oncology nurses. A national expert panel facilitated both phases in terms of carefully select items from the PRO-CTCAE library.

**Results:**

Phase one identified 18 symptoms and phase two, three additional ones, leading to the inclusion of 21 PRO-CTCAE symptoms in the final PRO tool. Since PRO-CTCAE also contains one to three sub-questions on the frequency, severity, and interference with daily activities of symptoms, there were 44 potential items.

**Conclusions:**

This study describes taking a multi-method approach to selecting items from the PRO-CTCAE library for use in a population of women with endometrial or ovarian cancer undergoing chemotherapy. By systematically combining diverse approaches, we carefully selected 21 clinically relevant symptoms covered by 44 items in the PRO-CTCAE library. Future studies should investigate the psychometric properties of this PRO tool for women with endometrial or ovarian cancer.

**Supplementary Information:**

The online version contains supplementary material available at 10.1186/s41687-023-00611-w.

## Abstract (highlights)

Women undergoing chemotherapy for endometrial or ovarian cancer experience a variety of symptoms and side effects associated with the disease and its treatment. One way to strengthen patient involvement during the treatment trajectory is to use patient-reported outcomes (PROs). Since PROs represent the patient’s response to inquiries about their current level of health, they should be tailored to the specific disease. As a result, we developed a PRO tool that targets women undergoing chemotherapy for endometrial or ovarian cancer. We conducted a comprehensive literature search and used various methods to map symptoms and side effects. Furthermore, discussions with and feedback from a patient advisory board, focus group interviews with oncologists and specialized oncology nurses, and advice from a gynecological national expert panel that we established helped us determine which symptoms a PRO tool for this patient group should include. This led to the inclusion of 21 symptoms in the final PRO tool, which is currently being tested. The selection process we applied may be generalizable for use in selecting PROs for other cancer populations.

**Supplementary Information:**

The online version contains supplementary material available at 10.1186/s41687-023-00611-w.

## Background

Endometrial and ovarian cancer account for almost 8% of all new cases of cancer specific to women worldwide each year [1]. Endometrial cancer is the sixth most common cancer in women, while ovarian cancer is the eighth [1]. Ovarian cancer is more lethal than endometrial cancer because ovarian cancer is often diagnosed at an advanced stage due to vague symptoms [2]. The specific diagnosis and stage of the disease determine treatment of endometrial and ovarian cancer, which may consist of a combination of surgery and chemotherapy [2–4]. Paclitaxel and carboplatin in combination, every three weeks, either adjuvant or neo-adjuvant, is the standard oncological treatment for advanced ovarian cancer [2, 5]. Adjuvant chemotherapy may be recommended for women with high-risk endometrial cancer [6, 7]. Following treatment, the women experience various disease- and treatment-related symptoms (e.g., bloating, sensory neuropathy, and constipation) [7–10], resulting in impaired quality of life and an increase in psychological burden that necessitate careful management [11, 12].

In daily oncological practice and clinical trials, a clinician reports the toxicities related to chemotherapy using Common Terminology Criteria of Adverse Events (CTCAE®), a standard measurement system for grading symptomatic adverse events [13, 14]. The National Cancer Institute created the PRO-CTCAE item library in response to healthcare providers frequently underestimating symptomatic adverse events compared to what patients experience and report [14, 15]. Based on contributions from patients and linguistically validated in more than 30 languages, including Danish, the PRO-CTCAE library reflects patient-reported adverse events in oncology and clinical trials [14, 16, 17] and contains 124 items covering 78 symptomatic adverse effects [14, 16, 17]. Relevant item sets targeting specific groups can be extracted by selecting symptomatic adverse events from the library [14, 17]. PRO-CTCAE gives patients the opportunity to respond to up to three questions related to each symptom to evaluate frequency, severity, and interference with daily activities [17, 18]. As a result, PRO-CTCAE includes adverse events with one, two, or three attributes [17, 18]. Currently, PROs are widely used and recommended by the U.S. Food and Drug Administration (FDA) [19, 20], and can also be collected electronically (ePROs). The systematic application of ePROs shows promising potential, for example, due to its ability to improve patient-clinician communication, patient engagement and satisfaction, quality of life, and possibly survival [21–25].

Recently, the use of ePRO was tested in the post-treatment follow-up of women with ovarian cancer [26] but remains to be tested in an endometrial or ovarian cancer population undergoing active taxane-platinum-based chemotherapy. In 2017, the Fifth Ovarian Cancer Consensus Conference expressed the need for context-specific PROs that reflect the patient population with ovarian cancer [27]. According to the most recent ESMO Clinical Practice Guideline [28], outcomes to be assessed in a routine clinical care setting must be meaningful in the target population and clinically actionable. Still, PRO tools must be created and adapted to specific patient populations to allow for appropriate, rapid monitoring of symptoms that patients may experience while undergoing chemotherapy [29]. As a result, the objective of the present study was to identify items in the PRO-CTCAE library for use in a targeted PRO tool for a population of women with endometrial or ovarian cancer undergoing chemotherapy with taxanes (paclitaxel or docetaxel) in combination with carboplatin.

## Material and methods

Using a combination of qualitative and quantitative methodologies, we applied a multi-methods approach to develop a targeted PRO tool for women with endometrial or ovarian cancer undergoing chemotherapy [30]. Inspired by similar research [31–33], we conducted a two-phase study where the outcomes in phase one guided phase two [31]. The symptoms were selected based on items in the PRO-CTCAE library [17] and emphasis was placed on symptoms that are either preventable or actionable for patients and healthcare professionals during treatment.

Phase one included a comprehensive literature search; a summary of phase III clinical studies and the toxicities of the relevant chemotherapeutics; and preliminary discussions in a patient advisory board comprising women with a history of gynecological cancer (n = 5). Patient and public involvement represent an essential contribution to this study and is the reason why the first international and evidence-based guidelines for patient and public involvement reporting in research, the Guidance for Reporting Involvement of Patients and the Public (GRIPP2) checklist (short version), was used [34] (Additional file 1: Table S1).

Phase two included two focus groups with specialized oncology nurses (n = 4) and senior gynecological oncologists (n = 4); an additional literature search for systematic reviews and meta-analyses; and several rounds of presentations and discussions with the patient advisory board.

**Fig. 1:**
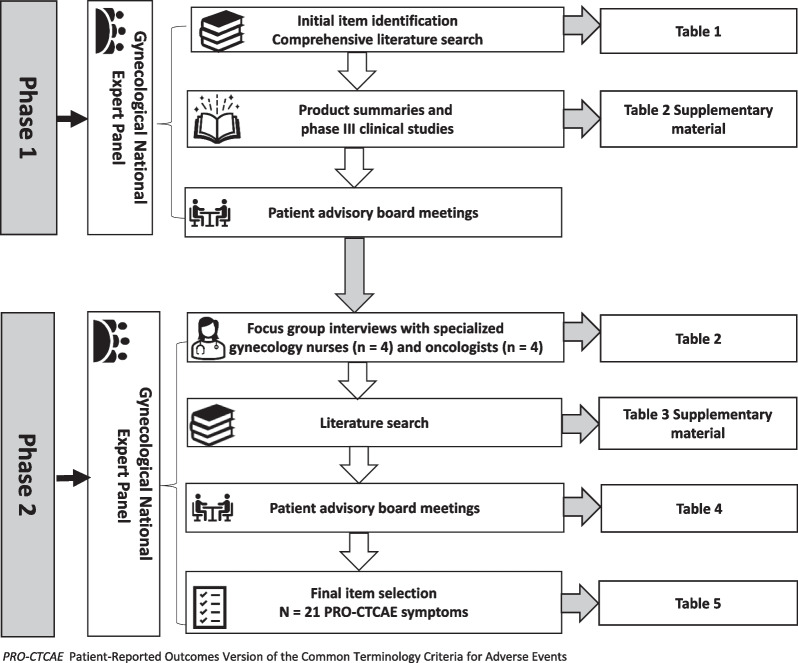
Item selection process

For the interviews, we applied convenience sampling and the sample size estimation was based on information power [35]. Participants were eligible if they had at least two years of experience in gynecological cancer. Due to the homogeneity of the participants we chose to do focus group interviews and they lasted about 45 min, and were audio recorded and transcribed verbatim. We emphasized group interaction and gaining a deeper understanding of the perspectives of the healthcare professionals on the most burdensome symptoms the patients with endometrial or ovarian cancer experienced [36]. After the interviews, the first author transcribed the audio recordings to determine which symptoms the healthcare professionals mentioned and considered significant. An additional comprehensive literature search was carried out to provide comparative knowledge to complement the results gathered in phase one. Figure 1 illustrates the item selection process.

### National expert panel

A multidisciplinary gynecological national expert panel was established to discuss and facilitate the selection of items throughout the entire selection process. Its four members comprised an associate professor and expert in nursing and symptom science; a professor and senior oncologist specializing in PRO and patient involvement; a professor in gynecologic cancer surgery and experienced PRO researcher; and a Ph.D. student experienced in gynecological cancer.

### Phase one

#### Initial item identification

The comprehensive literature search was conducted between February 2021 and March 2021 in the databases PubMed, Embase, and Web of Science to identify the relevant literature outlining symptoms associated with endometrial and ovarian cancer. The search was limited to English-language articles published between March 2011 and March 2021. We combined the keywords ovarian cancer, endometrial cancer, fallopian tube cancer, peritoneal cancer, chemotherapy, adverse events, and symptoms with the Boolean operators AND/OR in various combinations. To identify papers not found in the databases, we also screened reference lists in relevant publications and searched for citations in key papers.

#### Product information and phase III clinical studies

We reviewed the summary of product characteristics from the European Medicines Agency and FDA regarding toxicity related to the taxanes paclitaxel and docetaxel in combination with carboplatin [37, 38]. The toxicities were systematically mapped by following the hierarchy and terminology as described by the Medical Dictionary for Regulatory Activities (MedDRA)(39). The hierarchical structure allows flexible data retrieval and presentation [39]. We also identified phase III clinical studies documenting the toxicity of the relevant chemotherapeutics. We included toxicity that was given as very common (> 10%) and that may affect more than 1 in 10 for each chemotherapeutic. Additional file 2: Table S2 presents the results.

#### Patient advisory board

Following convenience sampling, a voluntary patient advisory board comprising five women with a history of endometrial or ovarian cancer was recruited in February and March 2021 via a closed online network group provided by a patient association for women with gynecological cancer. The women were invited to be research partners in a study. Following an initial conversation, interested women contacted the first author if they wished to participate on the board. Meetings were held online every two to three months. The board’s main objectives were to clarify and include the patient perspective in a Ph.D. study. However, because this study was an important part of other Ph.D. studies, the board was told at their first meeting about the item selection process and the 14 overall symptom categories in the PRO-CTCAE library [17]. The purpose of the patient advisory board in the current study was to clarify selection of the most appropriate symptoms, as well as to discuss preliminary and final results.

### Phase two

#### Literature search

A comprehensive literature search was carried out to provide comparative knowledge to supplement the results gathered in phase one and to explore knowledge on specific symptoms not fully covered in the core outcome set: anxiety and depression, insomnia, cognitive impairment, and sexuality. Our search used the following terms: anxiety, depression, cognitive impairment, platinum, taxane-based, chemotherapy, and sexuality in combination with the Boolean operators AND/OR. The search was limited to systematic reviews, meta-analyses, and females but was not exhaustive since the aim was to ensure the most up-to-date results by supplementing and synthesizing the most recent high-quality research on the specific symptoms from November 2015 to November 2021.

#### Focus group interviews

Using a purposive sampling strategy [40], healthcare professionals (specialized oncology nurses and senior gynecological oncologists) were recruited from a university hospital treating women with gynecological cancer. Two separate focus group interviews, one for each group, took place in November 2021 conducted and moderated by the first author. The purpose of the focus group interviews was to identify the type and characteristics of symptoms that the healthcare professionals saw as the most burdensome and pronounced for women with endometrial or ovarian cancer undergoing treatment with taxanes and carboplatin. Furthermore, the purpose was to confirm and expand on the PRO-CTCAE symptoms selected in phase one. The healthcare professionals were then subsequently asked to share their perspectives and insights on each of the symptoms. The interviews were conducted using a structured interview guide.

## Results

### Phase one

#### Initial item identification

We identified two reviews recommending a core outcome set: Reeve et al. [41], who recommended 12 core symptoms for use in adult cancer treatment trials, and Donovan et al. [42], who recommended using nine additional symptoms and quality-of-life domains in ovarian cancer treatment trials. We also identified two studies by King et al. [43, 44], who developed the questionnaire Measure of Ovarian Symptoms and Treatment (MOST), measuring the symptom benefit during chemotherapy for recurrent ovarian cancer. Webster et al. [45] conducted a pilot study aimed to assess user satisfaction using a PRO questionnaire for patients with gynecological cancer undergoing chemotherapy. We decided not to include the items identified in MOST because the target group was different (recurrent cancer versus taxane-based first-line chemotherapy). Moreover, Webster et al. [45] study had a broad scope that included all types of gynecological cancer and used Reeve et al. [41] 12 core symptoms but insufficiently described how the remaining symptoms were selected. As a result, after discussing the issue, the gynecological national expert panel decided to include Reeve et al. [41] and Donovan et al. [42] two core outcome sets as central components in our selection process. Our core symptom set ultimately comprised 18 PRO-CTCAE items (Table 1).

**Table 1:** Core outcome set and corresponding PRO-CTCAE items

MedDRA system organ classes^1^	Core outcome sets^2,3^	Corresponding PRO-CTCAE items^4^
Gastrointestinal disorders	➝	
	Constipation^2^	Constipation
	Diarrhea^2^	Diarrhea
	Nausea^2^	Nausea
	Bloating^3^	Bloating
	Vomiting^3^	Vomiting
	Cramping^3^	Bloating/abdominal pain
	Indigestion^3^	Diarrhea, constipation, abdominal pain
General disorders and administration site conditions	➝	
	Pain^2^	Abdominal pain
	Abdominal pain^3^	Abdominal pain
	Fatigue^2^	Fatigue
Metabolism and nutrition disorders	➝	
	Anorexia (appetite loss)^2^	Decreased appetite
	Weight gain^3^	N/A
	Weight loss^3^	Decreased appetite
Nervous system disorders	➝	
	Sensory neuropathy^2^	Numbness & tingling
Psychiatric disorders	➝	
	Anxiety (includes worry)^2^	Anxious
	Cognitive problems^2^	Concentration, memory
	Depression (includes sadness)^2^	Discouraged, sad
	Fear of recurrence/disease progression^3^	N/A
	Insomnia^2^	Insomnia
Reproductive system and breast disorders	➝	
	Sexual dysfunction^3^	Decreased libido
		Vaginal dryness
Respiratory, thoracic, and mediastinal disorders	➝	
	Dyspnea^2^	Shortness of breath
Unique symptoms in total	21	18

*MedDRA *Medical Dictionary for Regulatory Activities,* PRO-CTCAE* Patient-Reported Outcomes Version of the Common Terminology Criteria for Adverse Events

^1^Medical Dictionary for Regulatory Activities (MEDDRA) [Internet]. Available from: https://www.meddra.org/ [39], ^2^Reeve et al. [41], ^3^Donovan et al. [42],^4^PRO-CTCAE. PRO-CTCAE® Measurement System website [Internet]. Available from: https://healthcaredelivery.cancer.gov/pro-ctcae [17]

#### Product information and phase III clinical studies

The product summary from the European Medicines Agency [37] and FDA [38] and the phase III clinical studies (n = 3) [46–48] were systematically registered according to MedDRA [39] and aligned with the associated PRO-CTCAE symptom, yielding 18 symptoms (Additional file 2: Table S2).

## Phase two

### Focus group interviews

Four gynecological oncology nurses and four senior gynecological oncologists participated in two separate focus group interviews. Overall, both groups had more than 11 years of working experience on average and more than 8 years of experience in oncology, on average. Table 2 shows the characteristics of the nurses and the oncologists.

**Table 2:** Characteristics of nurses and oncologists

	Nurses (n = 4) years	Oncologists (n = 4) years
Age		
Mean (range)	45.0 (39–59)	63.0 (54–70)
Average years since graduation (range)	11.50 (9–15)	36.50 (25–45)
Average years in the oncological specialty (range)	8.25 (4–11)	23.75 (10–26)

Both groups listed the symptoms they thought were important in terms of the specific population, with oncologists primarily emphasizing physical symptoms and then psychological symptoms. The nurses, on the other hand, believed that fear of recurrence, fear of death, anxiety, and emotional symptoms were extremely important, though both groups listed physical and psychological symptoms as burdensome. Both groups identified neurotoxicity as the main difference between the docetaxel and paclitaxel treatment schedules. Table 3 lists the symptoms as identified by each group.

**Table 3:** Symptoms outlined healthcare professionals in the focus group interviews

Identified symptoms	Specialized oncology nurses (n = 4)	Senior gynecological oncologists (n = 4)
Fatigue	x	x
Neuropathy	x	x
Nausea	x	x
Dyspnea	x	
Constipation	x	x
Symptoms of the flu (sub-febrile, muscle pain)	x	x
Mucositis	x	x
Vaginal dryness	x	x
Myelosuppression (anemia, febrile neutropenia, and thrombocytopenia)	x	x
Hearing loss/tinnitus	x	x
Concerns for family and children	x	
Anxiety	x	
Depression	x	x
Fear of death	x	
Insomnia	x	
Weight loss	x	x
Alopecia	x	x
Nail changes	x	x
Diarrhea	x	x
Abdominal pain		x
Sensitive mucous membranes	x	x
Altered body image		x
Fear of recurrence	x	x
Memory		x
Post-traumatic stress disorder		x
Concentrating		x

A preliminary PRO tool based on the literature search, product summaries, and information from the clinical phase III trials was discussed at the end of both focus group interviews, with the participants elaborating upon and confirming the selected PRO-CTCAE symptoms.

### Comprehensive literature search

The search revealed systematic reviews (n = 4) [11, 49–51] and a meta-analysis (n = 1) [52]. The populations mainly comprised ovarian cancer (n = 3) [11, 50, 51], uterine cancer (n = 1) [49], and breast cancer survivors (n = 1) [52] (Additional file 3: Table S3). The meta-analysis, which included breast cancer survivors only [52], investigated depression and cognitive impairment caused by taxane-based chemotherapy. We included this information due to treatment similarities with the gynecological population as there is a lack of research in this population.

### Patient advisory board meeting

The members of the board had a mean age of 68.4 years and had all received platinum-based chemotherapy as their first-line treatment. Table 4 outlines the characteristics of the patient advisory board.

**Table 4:** Characteristics of the patient advisory board

*Age*	
Mean (years (range))	68.4 (59–77)
*Diagnosis*	
Ovarian cancer	4
Endometrial cancer	1
*Treatment status*	
Active oncological	4
Follow-up	1
Average years since diagnosis (years (range))	4.8 (1–8)
*Relationship status*	
Married	3
Single	2
*Highest completed education*	
Short	1
Medium	4
*Employment status*	
Working part-time	1
Retired	4

Five patient advisory board meetings were held from April to December 2021. The board emphasized the importance of including gastrointestinal symptoms, such as cramping, bloating, ileus, and constipation because they were seen as important and burdensome but were rarely addressed in clinical practice. However, the board agreed that including bloating, constipation, and abdominal pain was sufficient because patients had difficulty distinguishing between them. The board, which highlighted the significance of including explicitly sexual symptoms, discussed the sexual symptoms in the PRO-CTCAE library [17] but decided that including delayed orgasm, unable to have orgasm, and pain with sexual intercourse would be too specific and not suited to weekly monitoring, which is why they felt using decreased libido and vaginal dryness was better. They argued that because healthcare professionals frequently fail to mention these symptoms, future patients would likely benefit from a stronger emphasis on communicating about them. The board agreed that sexuality-related issues should be discussed more frequently and openly in clinical practice. The board also advised that symptoms related to the oral cavity should be included to promote more effective management of them, leading us to select mouth/throat sores, which are likewise outlined in the product summaries. Finally, aware of the risk of questionnaire fatigue, the gynecological national expert panel discussed the overall number of items. However, the board argued that the number of symptoms was appropriate because a patient would never experience all possible symptoms at once.

### Final item selection

The expert panel held several online meetings to discuss the results including pain as a symptom, but it was deemed too generic and was replaced with the disease-specific abdominal pain. Nearly all 12 symptoms identified by Reeve et al. [41] were chosen and matched with the associated PRO-CTCAE symptoms, resulting in 14 PRO-CTCAE symptoms in total. Cramping, indigestion, and sexual dysfunction, which were symptoms Donovan et al. [42] recommended, were either covered by an already selected symptom or matched to the corresponding PRO-CTCAE symptom [17] (Table 1). Furthermore, we decided not to incorporate fear of recurrence/disease progression and weight gain, because our target population primarily comprised women receiving first-line chemotherapy, making those two symptoms inappropriate for weekly monitoring. The expert panel also debated whether to include taste changes, rash, hair loss, and nail disorder since these symptoms were identified in the summary of applied chemotherapeutics and clinical phase III studies (Additional file 2: Table S2). To reduce the overall respondent burden, they were not included, also because these symptoms and the corresponding PRO-CTCAE symptom were seen as either too general or insufficient for weekly monitoring.

Since the expert panel agreed that sexuality and intimacy were highly important, the decision was made to include two PRO-CTCAE symptoms, decreased libido and vaginal dryness, since sexual health is an area that needs more attention in clinical practice [53, 54]. The panel discussed whether to include changes in body image as a symptom but doing so would necessitate the use of an additional item because the PRO-CTCAE library [17] does not contain it, leading us to decide against it since the questions would be asked inconsistently.

Finally, the literature search, product summaries, clinical phase III studies, focus group interviews, and patient advisory board discussions resulted in the inclusion of 21 symptoms covered by 44 items from the PRO-CTCAE library [17]. These symptoms appear to be the best compromise in terms of minimizing respondent burden while still covering all relevant symptoms. The 44 items were selected using a branching logic that incorporated sub-items examining frequency, severity, and interference with daily activities [17]. Table 5 summarizes the overall findings.

**Table 5:** Summary of results from the item selection process

MedDRA system organ classes^1^	Identified PRO-CTCAE symptoms based on two core outcome sets^2,3^	Identified symptoms from product summaries and phase III clinical studies^4,5,6^, aligned with matching PRO-CTCAE symptoms^7^	Final PRO-CTCAE symptoms^7^ included in the PRO tool
Gastrointestinal disorders			
	Constipation	Constipation	Constipation
	Diarrhea	Diarrhea	Diarrhea
	Nausea	Nausea	Nausea
	Bloating	Mouth/throat sores	Mouth/throat sores
	Vomiting	Vomiting	Vomiting
			Bloating
General disorders and administration site conditions			
	Fatigue	Fatigue	Fatigue
	Abdominal pain	General pain	Abdominal pain
Metabolism and nutrition disorders			
	Decreased appetite	Decreased appetite	Decreased appetite
Musculoskeletal and connective tissue disorders			
		Joint pain	Joint pain
		Muscle pain	Muscle pain
Nervous system disorders			
	Numbness & tingling	Numbness & tingling	Numbness & tingling
Psychiatric disorders			
	Anxious		Anxious
	Concentration		Concentration
	Discouraged		Discouraged
	Memory		Insomnia
	Insomnia		Memory
	Sad		Sad
Reproductive system and breast disorders			
	Sexual dysfunction		Decreased libido
	Vaginal dryness		Vaginal dryness
Respiratory, thoracic and mediastinal disorders			
	Shortness of breath	Shortness of breath	Shortness of breath
Skin and subcutaneous tissue disorders			
		Hair loss	
		Nail loss	
		Nail ridging	
		Nail discoloration	
		Rash	
Investigations			
		Taste changes	
Unique symptoms, total	18	18	21

*PRO-CTCAE* Patient-Reported Outcomes Version of the Common Terminology Criteria for Adverse Events, *MedDRA* Medical Dictionary for Regulatory Activities

^1^Medical Dictionary for Regulatory Activities (MedDRA) [Internet]. Available from: https://www.meddra.org/ (39), ^2^Reeve et al. [41], ^3^Donovan et al. [42], ^4^Du Bois et al.[48], ^5^Ozols et al. [47], ^6^Vasey et al. [46], ^7^PRO-CTCAE. PRO-CTCAE® Measurement System website [Internet]. Available from: https://healthcaredelivery.cancer.gov/pro-ctcae/ [17]

## Discussion

To the best of our knowledge, this study is the first to describe and develop a PRO tool that is suitable for use in clinical settings targeting women with endometrial and ovarian cancer undergoing chemotherapy with a taxane in combination with carboplatin. Since the PRO-CTCAE library provides flexibility in selecting items, it was used to determine the 21 symptoms in the PRO tool we developed [14, 17]. In line with this, a recent consensus guideline [3] recommends self-reported toxicity measurement instruments like PRO-CTCAE to be used in women with gynecological cancer, emphasizing the importance of our PRO tool.

Patient involvement in research is gaining more attention and can help identify relevant outcomes [55]. In this study, the patient advisory board endorsed the items chosen, confirming that they accurately reflect patient symptoms. Patients with gynecological cancer experience severe emotional and physical strain during chemotherapy that impairs their quality of life [56, 57]. A systematic review and meta-analysis of the prevalence of depression and anxiety in an ovarian cancer population found that 23% of women experience depression during treatment and 26% report anxiety [11]. Anxiety was also one of the top five most discussed topics in an online social media platform [58], underscoring the importance of recognizing it as a significant symptom to include. As recommended, we included five questions on psychological well-being since greater emphasis on this during treatment may further help and support patients [57]. Our primary focus was to develop a PRO tool that assessed symptoms that were either preventable or manageable during treatment since doing so may improve self-management. Ovarian-specific symptoms such as bloating, abdominal pain, and constipation are rarely addressed in clinical practice [59], and weekly assessment may prevent hospitalization and even treatment delays. Thus, apart from symptom identification, establishing a patient-clinician dialogue based on evidence-based symptom management in a multidisciplinary setting is crucial.

Applying a PRO tool designed specifically for this gynecological population allows us to learn more about the physical and mental well-being of patients between treatment cycles. The tri-weekly schedule in current clinical practice increases the risk that patients will be unable to recall significant symptoms, side effects, and fluctuations over time. According to one study [60], 61% of patients with ovarian cancer did not discuss their most concerning symptoms with their healthcare professionals, indicating that there is room for improvement. Using a PRO tool may reduce the patient’s burden because simple and direct questions in a weekly PRO can cover the most common symptoms [61]. Thus, adequately monitoring symptoms necessitates having clinicians trained in closely monitoring and following up on patient responses to guide optimal symptom interventions.

Our comprehensive search showed that other measures exist for women with gynecological cancer and recurrent ovarian cancer and that these measures could arguably have been used in our selection process. The measures differ, however, and our study aimed to develop a specific PRO tool targeting our population. Webster et al.’s [45] aim, in contrast, was to assess user satisfaction with a focused PRO questionnaire for patients with gynecological cancer; in other words, their study started where ours ended. For MOST T24 [44, 62] the aim was to measure symptom benefits during chemotherapy for recurrent ovarian cancer and focus on symptoms and well-being. Additional file 4: Table S4 outlines a comparison of the measures. When we compare the symptoms in our PRO tool to the results of King et al. [44, 62] and Webster et al.[45], we find that our symptoms are 52% similar to the symptoms identified by King et al. [44] and 86% similar to the symptoms identified by Webster et al. [45]. As a result, our PRO tool differs in that it is intended for weekly ePRO monitoring and is tailored mainly to patients with endometrial or ovarian cancer undergoing primarily first-line chemotherapy. The similarities between the PRO-CTCAE symptoms Webster et al. [45] selected and the ones in our PRO tool indicate that the symptoms in our PRO tool are appropriate. Again, Webster et al.’s [45] results demonstrate that a tool based on PRO-CTCAE items was acceptable to patients and clinicians, contained relevant content, and had a positive impact on clinical care. This reflects the growing interest in using questionnaires based on the PRO-CTCAE library to capture patient self-reported symptoms [32, 63, 64], and our study findings add to the understanding of this specific population. A future interventional study will allow patients to select only the symptoms they are experiencing and add other specific symptoms they may be experiencing. In a recent study Beesley et al. [65] explored follow-up monitoring of first-line treatment using the new MOST S26. Our PRO tool and MOST S26 [62, 65] can supplement one another to allow us to better understand the symptoms that occur during and after chemotherapy for the benefit of patients.

Martin et al. [10] found that the most bothersome symptoms reported by women treated for ovarian cancer were bloating, abdominal pain, and tiredness. Abdominal bloating is a well-known symptom among women with ovarian cancer [66], but to the best of our knowledge, no evidence-based guidelines for treatment or management exist. Closely monitoring it and its severity is important, which is why a weekly ePRO may be the key to determining the extent of the problem among women undergoing chemotherapy. One of the most common and distressing quality-of-life issues for female cancer survivors is sexual dysfunction [53]. This symptom is also common among women treated for ovarian cancer, with nearly half of all women experiencing some kind of sexual dysfunction [67]. The appropriate timing for addressing sexual function and concerns about sexuality is arguably around the time of diagnosis and when treatment begins [53]. For these reasons, we included decreased libido and vaginal dryness in the hope that early identification, support, and attention can improve sexual health during chemotherapy. The patient advisory board’s thorough discussion of this topic and the large body of research on it confirmed and strengthened our decision to emphasize it more heavily in clinical practice.

Some patients with ovarian cancer may be treated with bevacizumab in combination with taxane and carboplatin, but new treatment options are constantly evolving [3]. However, we did not include bevacizumab-specific symptoms in our PRO tool because they are either covered by other items or are clinical parameters (e.g., hypertension and proteinuria) or acute (bleeding) [68], which means addressing them in a PRO tool is not relevant. Docetaxel and carboplatin are the standard of care in some Danish hospitals for patients who are not enrolled in clinical trials, while paclitaxel and carboplatin are the standard of care for advanced endometrial cancer, which is why we included both taxanes while developing our PRO tool. In many ways, the two regimens are similar, except that paclitaxel causes more neurotoxicity [46].

One could argue that we should have included more symptoms or other relevant symptoms, such as nail disorders and taste changes but they are normally recognized in clinical practice before prescribing chemotherapy, which is why they are not included in the final PRO tool. Another possible aspect to consider is whether lower extremity lymphedema should be monitored weekly in the PRO tool since it can occur especially in the endometrial population undergoing surgery [69]. We decided not to do so for several reasons, including a lack of standardized quantitative measurements for optimal lower extremity lymphedema [70], an expected decrease in the prevalence of lymphedema in the future due to improved surgical methods involving sentinel node mapping [71], and a desire to keep the number of symptoms as low as possible to reduce the respondent burden. Nonetheless, giving patients the option of adding free-text symptoms will allow us to suitably modify the PRO tool if they are reported often. Our PRO platform thoroughly describes lower extremity lymphedema in plain language as an additional symptom and recommends self-monitoring management, but the need to add this symptom to our tool should be examined further. We believe the present PRO tool is relevant and contains the most predominant symptoms in the gynecological population undergoing taxane and platinum-based chemotherapy. Our PRO tool has the potential to contribute to adequate and timely symptom identification, laying the foundation for a valuable patient dialogue.

### Strengths and limitations

To ensure stringency, the PRO tool is based only on symptoms from the validated PRO-CTCAE library [17]. We value this as a strength, as the format of the items and the response categories are similar, making them easy to understand and respond to. Our multi-method approach allowed us to combine various methods, which, in conjunction with our systematic approach, provided a PRO tool ready to use in a targeted population. The patient advisory board’s repeated involvement with the selection process is a significant strength and the fact that they had all previously been treated with taxane and platinum-based chemotherapy means they represented the future target population. Capturing patient voices helped us ensure the content validity of our PRO tool. It would have been beneficial to record feedback more formally from the board as opposed to solely taking notes on their work. Another limitation is that we did not conduct individual or focus group interviews, nor did we include patient representatives on the national expert panel for gynecology, which may have strengthened the content validity further. Our PRO tool is currently being tested in an intervention study, with results expected within a year. The results of this study will also include a systematic assessment of this patient population. The use of the GRIPP2 checklist for reporting patient and public involvement is a strength since it improves the overall quality and transparency of our study [34]. The collaboration and discussions in the national expert panel and the focus group interviews with clinical experts also strengthened the content validity. The detailed and diverse perspectives provided by the focus group interviews are regarded as a strength. Given the aim of the study, the use of focus group interviews with specialized nurses and oncologists, information power with eight participants was considered sufficient [35]. Using a chart review may have strengthened our study, but we decided against it due to Tolstrup et al.’s [32] discovery of a high level of agreement between the results of chart audits and the literature and product information. Unfortunately, most of the literature included in this study is based on ovarian cancer, limiting use of the PRO tool in endometrial cancer. However, women with ovarian or endometrial cancer receiving taxane and platinum-based chemotherapy will most likely experience similar treatment-related symptoms, which justifies the development of additional PRO tools for both diagnoses.

## Conclusion

This study describes a multi-method approach to selecting items from the PRO-CTCAE library for use in women undergoing chemotherapy with a taxane and carboplatin for endometrial or ovarian cancer. A multi-method approach was used to carefully select 21 clinically relevant PRO-CTCAE symptoms, which were covered by 44 items. This multi-method sequential approach to selecting relevant symptoms for inclusion in a PRO tool for a specific gynecological population may be transferable to other groups of patients with cancer. Future studies should investigate the psychometric properties of our PRO tool for women with endometrial or ovarian cancer.

## Supplementary Information

**Additional file 1.** GRIPP2 checklist.

**Additional file 2.** Product information and phase III clinical studies.

**Additional file 3.** Overview of Systematic reviews and meta-analysis.

**Additional file 4.** Comparison of symptoms in various questionnaires and outcome sets.

## Data Availability

Danish law precludes making the datasets generated and/or analyzed during the current study publicly available, but they can be made available by the corresponding author upon reasonable request.

## Abbreviations

CTCAE: Common Terminology Criteria of Adverse Events
FDA: U.S. Food and Drug Administration
ePRO: electronic Patient-Reported Outcome
GRIPP: Guidance for reporting involvement of patients and the public
MedDRA: Medical Dictionary for Regulatory Activities
MOST: Measure of Ovarian Symptoms and Treatment
PRO: Patient-Reported Outcome
PRO-CTCAE: Patient-Reported Outcomes version of the Common Terminology Criteria for Adverse Events
