# Supplementary material for: A multi-method approach to selecting PRO-CTCAE symptoms for patient-reported outcome in women with endometrial or ovarian cancer undergoing chemotherapy
Source: J Patient Rep Outcomes. 2023 Jul 18;7:72. doi: 10.1186/s41687-023-00611-w (PMC10354345; doi:10.1186/s41687-023-00611-w)
Supplement: Supplementary file 1 — Additional file 1. GRIPP2 checklist. [file 41687_2023_611_MOESM1_ESM.docx]

**Additional file 1: GRIPP2 checklist** [1]

| Section and topic | Item | Reported on page No. |
| --- | --- | --- |
| 1: Aim | Report the aim of PPI in the study | 5 |
| 2: Methods | Provide a clear description of the methods used for PPI in the study | 5 |
| 3: Study results | Outcomes—Report the results of PPI in the study, including both positive and negative outcomes | 10 |
| 4: Discussion and conclusions | Outcomes—Comment on the extent to which PPI influenced the study overall. Describe positive and negative effects | 12–15 |
| 5: Reflections/critical perspective | Comment critically on the study, reflecting on the things that went well and those that did not, so others can learn from this experience | 15–16 |

*GRIPP2* Guidance for Reporting Involvement of Patients and the Public, short version, *PPI* patient and public involvement

[1] S. Staniszewska et al, ‘GRIPP2 reporting checklists: Tools to improve reporting of patient and public involvement in research’, *BMJ*, vol. 358, 2017, doi: 10.1136/bmj.j3453.
