# Supplementary material for: A multi-method approach to selecting PRO-CTCAE symptoms for patient-reported outcome in women with endometrial or ovarian cancer undergoing chemotherapy
Source: J Patient Rep Outcomes. 2023 Jul 18;7:72. doi: 10.1186/s41687-023-00611-w (PMC10354345; doi:10.1186/s41687-023-00611-w)
Supplement: Supplementary file 2 — Additional file 2. Product information and phase III clinical studies. [file 41687_2023_611_MOESM2_ESM.docx]

|  |  | **Summary of product characteristics** | | | | | | **Clinical phase III studies** | | |  |  |  |  |
| --- | --- | --- | --- | --- | --- | --- | --- | --- | --- | --- | --- | --- | --- | --- |
| **MedDRA system organ class (listed alphabetically)** | **Symptoms very common (>10 %)** | **Docetaxel EMA** | **Docetaxel FDA** | **Paclitacel EMA** | **Paclitaxel FDA** | **Carboplatin EMA** | **Carboplatin FDA** | **Du Bois et al.** [1] | **Ozols et al.** [2] | **Vasey et al.** [3] | **Symptoms originating from product resumes and phase III clinical studies aligned with PRO-CTCAE** | **Reeve et al.** [4] **Core outcome set** | **Donovan et al.** [5] **Core outcome set** | **Final PRO-CTCAE symptoms included** |
| **Blood and lymphatic system disorders** | Neutropenia | x | x | x | x | x | x |  |  | x |  |  |  |  |
|  | Anemia | x | x | x |  | x | x |  |  | x |  |  |  |  |
|  | Thrombo-cytopenia | x |  | x | x | x | x |  | x | x |  |  |  |  |
|  | Febrile neutropenia (common) |  |  |  | x |  |  | x |  |  |  |  |  |  |
|  | Bleeding/  Hemorrhage |  |  | x |  |  | x |  |  |  |  |  |  |  |
|  | Myelosuppresion |  |  | x |  |  |  |  |  |  |  |  |  |  |
|  | Placelets |  |  |  |  |  |  | x |  |  |  |  |  |  |
|  | Hematological |  |  |  |  |  |  | x |  |  |  |  |  |  |
|  | Granulocytopeni |  |  |  |  |  |  |  | x |  |  |  |  |  |
|  | Leucopenia |  | x | x |  | x | x | x | x |  |  |  |  |  |
|  | Transfusions |  |  |  |  |  | x |  |  |  |  |  |  |  |
| **Cardiac disorders** | Cardiovascular |  |  |  |  |  | x |  |  |  |  |  |  |  |
|  | Cardiac toxicity |  |  |  |  |  |  | x |  |  |  |  |  |  |
| **Ear and labyrinth disorders** | Ototoxicity |  |  |  |  |  | x | x |  |  |  |  |  |  |
|  | Impaired hearing |  |  |  |  | x |  |  |  |  |  |  |  |  |
| **Gastro-intestinal disorders** | Nausea | x | x | x | x | x | x | x |  | x | Nausea | **x** |  | **Nausea** |
|  | Stomatitis | x | x |  |  |  |  | x |  | x | Mouth/ throat sores |  |  | **Mouth/ throat sores** |
|  | Vomiting | x | x | x | x | x | x | x |  | x | Vomiting |  | **x** | **Vomiting** |
|  | Diarrhea | x | x | x | x | x |  | x |  | x | Diarrhea | **x** |  | **Diarrhea** |
|  | Constipation |  |  |  |  |  |  | x |  | x | Constipation | **x** |  | **Constipation** |
|  | Mucositis |  |  |  |  |  | x | x |  |  | Mouth/ throat sores |  |  |  |
|  | Mucosal inflammation |  |  | x |  |  |  |  |  |  | Mouth/ throat sores |  |  | **Vaginal dryness** |
|  | Abdominal pain |  |  |  |  | x |  |  |  | x |  |  | **x** | **Abdominal pain** |
|  | Gastrointestinal/  indegestion |  |  |  |  |  |  |  | x |  | Nausea, vomiting, consti-pation, diarrhea |  | **x** |  |
|  | Bloating |  |  |  |  |  |  |  |  |  |  |  | **x** | **Bloating** |
|  | Cramping |  |  |  |  |  |  |  |  |  |  |  | **x** |  |
| **General disorders and administra-tion site conditions** | Astenia | x | x | x | x | x | x |  |  |  | Fatigue |  |  |  |
|  | Fluid retention | x | x |  |  |  |  |  |  |  |  |  |  |  |
|  | Pain | x |  |  |  |  | x |  | x |  | General pain |  |  |  |
|  | Fatigue |  |  |  |  |  |  |  |  | x | Fatigue | x |  | **Fatigue** |
| **Immune system disorders** | Hypersentivity |  |  | x | x | x | x | x |  | x |  |  |  |  |
|  | Allergic |  |  |  |  |  | x |  |  |  |  |  |  |  |
|  | Minor hypersensitivity reactions (mainly flushing and rash) |  |  | x |  |  |  |  |  |  |  |  |  |  |
| **Metabolism and nutrition disorders** | Infections | x | x | x | x |  | x | x |  |  |  |  |  |  |
|  | Metabolic |  |  |  |  |  |  |  | x |  |  |  |  |  |
|  | Decreased appetite |  |  |  |  |  |  |  |  |  |  |  |  | **Decreased appetite** |
|  | Anorexia | x |  |  |  |  |  |  |  |  | Decreased appetite | **x** |  |  |
|  | Weight gain |  |  |  |  |  |  |  |  |  |  |  | **x** |  |
|  | Weight loss |  |  |  |  |  |  |  |  |  |  |  | **x** |  |
| **Musculo-skeletal and connective tissue disorders** | Myalgia |  |  | x | x |  |  | x |  | x | Muscle pain |  |  | **Muscle pain** |
|  | Atralgia |  |  | x | x |  |  | x |  | x | Joint pain |  |  | **Joint pain** |
|  | Pain |  |  |  |  |  | x |  |  |  |  | x |  |  |
| **Nervous system disorders** | Peripheral sensory neuropathy | x |  |  |  |  | x |  |  | x | Numbness & tingling |  |  | **Numbness & tingling** |
|  | Neurosensory |  | x |  |  |  |  |  |  |  |  | x |  |  |
|  | Neuromotor |  | x |  |  |  |  |  |  |  |  |  |  |  |
|  | Neurotoxicity |  |  | x | x |  |  |  |  |  |  |  |  |  |
|  | Peripheral mortor neuropathy (common) |  |  |  |  |  |  | x |  | x |  |  |  |  |
|  | Central neurotoxicity |  |  |  |  |  | x | x |  |  |  |  |  |  |
|  | Neurologic |  |  |  |  |  |  |  | x |  |  |  |  |  |
| **Reproductive system and breast disorders** | Genitourinary |  |  |  |  |  | x |  | x |  |  |  |  |  |
|  | Sexual dysfunction |  |  |  |  |  |  |  |  |  |  |  | **x** | **Decreased libido** |
| **Respiratory, thoracic and mediastinal disorders** | Shortness of breath |  |  |  |  |  |  |  | x |  | Shortness of breath |  |  | **Shortness of breath** |
|  | Respiratory |  |  |  |  |  | x |  |  |  |  |  |  |  |
|  | Pulmonary |  | x |  |  |  |  |  |  |  |  |  |  |  |
|  | Dyspnea |  |  |  |  |  |  |  |  |  |  | **x** |  |  |
| **Psychiatric disorders** | Insomnia |  |  |  |  |  |  |  |  |  |  | **x** |  | **Insomnia** |
|  | Cognitive problems |  |  |  |  |  |  |  |  |  |  | **x** |  | **Concentra-tion** |
|  |  |  |  |  |  |  |  |  |  |  |  |  |  | **Memory** |
|  | Anxiety (includes worry) |  |  |  |  |  |  |  |  |  |  | **x** |  | **Anxious** |
|  | Depression (includes sadness) |  |  |  |  |  |  |  |  |  |  | **x** |  | **Sad** |
|  |  |  |  |  |  |  |  |  |  |  |  |  |  | **Discouraged** |
|  | Fear of recurrence/ disease progression |  |  |  |  |  |  |  |  |  |  |  | **x** |  |
| **Skin and subcuta-neous tissue disorders** | Alopecia | x | x | x | x |  | x | x | x | x | Hair loss |  |  |  |
|  | Nail disorder |  | x |  |  |  |  |  |  | x | Nail loss, nail ridging, nail discloration |  |  |  |
|  | Skin reaction | x | x |  |  |  |  |  |  |  | Rash |  |  |  |
| **Vascular disorders** | Hypotension |  |  | x |  |  |  |  |  |  |  |  |  |  |
| **Investiga-tions** | Changes in your sense of taste |  |  |  |  |  |  |  |  | x | Taste changes |  |  |  |
|  | Creatinine renal clearance decreased |  |  |  |  | x |  |  |  |  |  |  |  |  |
|  | Increased blood urea |  |  |  |  | x | x |  |  |  |  |  |  |  |
|  | Increased blood alkaline phosphatase |  |  |  |  | x |  |  |  |  |  |  |  |  |
|  | Increased aspartate aminotransferase |  |  |  |  | x |  |  |  |  |  |  |  |  |
|  | Abnormal liver function test |  |  |  |  | x |  |  |  |  |  |  |  |  |
|  | Decreased blood sodium |  |  |  |  | x | x |  |  |  |  |  |  |  |
|  | Decreased blood potassium |  |  |  |  | x | ´x |  |  |  |  |  |  |  |
|  | Decreased blood calcium |  |  |  |  | x | x |  |  |  |  |  |  |  |
|  | Decreased blood magnesium |  |  |  |  | x | x |  |  |  |  |  |  |  |
|  | SGOT elevations |  |  |  |  |  | x |  |  |  |  |  |  |  |
| **Total** |  | 15 | 16 | 19 | 13 | 20 | 28 | 19 | 10 | 18 | **18** | **12** | **9** | **21** |

*MedDRA* Medical Dictionary for Regulatory Activities, *EMA* European Medcines Agency, *FDA* U.S. Food and Drug Administration, *PRO-CTCAE* Patient-Reported Outcomes Version of the Common Terminology Criteria for Adverse Events

**References:**

[1] A. du Bois et al, ‘A randomized clinical trial of cisplatin/paclitaxel versus carboplatin/paclitaxel as first-line treatment of ovarian cancer’, *J. Natl. Cancer Inst.*, vol. 95, no. 17, pp. 1320–1330, 2003, doi: 10.1093/jnci/djg036.

[2] R. F. Ozols et al, ‘Phase III trial of carboplatin and paclitaxel compared with cisplatin and paclitaxel in patients with optimally resected stage III ovarian cancer: A Gynecologic Oncology Group study’, *J. Clin. Oncol.*, vol. 21, no. 17, pp. 3194–3200, 2003, doi: 10.1200/JCO.2003.02.153.

[3] P. A. Vasey et al, ‘Phase III randomized trial of docetaxel-carboplatin versus paclitaxel-carboplatin as first-line chemotherpy for ovarian carcinoma’, *J. Natl. Cancer Inst.*, vol. 96, no. 22, pp. 1682–1691, 2004, doi: 10.1093/jnci/djh323.

[4] B. B. Reeve et al, ‘Recommended patient-reported core set of symptoms to measure in adult cancer treatment trials’, *J. Natl. Cancer Inst.*, vol. 106, no. 7, 2014, doi: 10.1093/jnci/dju129.

[5] K. A. Donovan et al, ‘Recommended patient-reported core set of symptoms and quality-of-life domains to measure in ovarian cancer treatment trials’, *J. Natl. Cancer Inst.*, vol. 106, no. 7, pp. 10–13, 2014, doi: 10.1093/jnci/dju128.
