# Supplementary material for: A multi-method approach to selecting PRO-CTCAE symptoms for patient-reported outcome in women with endometrial or ovarian cancer undergoing chemotherapy
Source: J Patient Rep Outcomes. 2023 Jul 18;7:72. doi: 10.1186/s41687-023-00611-w (PMC10354345; doi:10.1186/s41687-023-00611-w)
Supplement: Supplementary file 3 — Additional file 3. Overview of Systematic reviews and meta-analysis. [file 41687_2023_611_MOESM3_ESM.docx]

**Additional file 3. Overview of Systematic reviews and meta-analysis**

| **Authors, years** | **Country** | **Population** | **Aim/purpose of the systematic review** | **Search databases** | **Types of studies included** | **The number of studies included** | **Summary of results** |
| --- | --- | --- | --- | --- | --- | --- | --- |
| Watts et al. (2015)(1) | United Kingdom | Ovarian cancer | To systematically review the literature regarding the prevalence of depression and anxiety in patients with ovarian cancer as a function of the treatment stage. | OVID MEDLINE, EMBASE, AMED, PsycINFO, CINAHL, and Web of Science | Cross-sectional (n=18), Longitudinal (n=7) | (n=24) | The prevalence of depression and anxiety in women with ovarian cancer is significantly greater than in the healthy female population. Further research is warranted to ensure psychological distress in ovarian cancer is not underdiagnosed and undertreated. |
| Alanazi et al. (2022)(2) | Saudi Arabia | Uterine cancer | To describe the sleep patterns among uterine cancer survivors and verify psychological and physical factors affecting their general qualities of life. | PubMed Cochrane Trial Register and ScienceDirect | RCT (n=5), Cohort study (n=3), cross-sectional (n=2) | (n=10) | 61% of uterine cancer survivors had poor sleep quality, and 81% have a decreased quality of life. |
| Palagini et al. (2021)(3) | Italy | Ovarian cancer | To systematically review the prevalence and management of insomnia and circadian sleep disorders in ovarian cancer | PubMed |  | (n=22) | Insomnia is a frequent symptom in more than half of patients with OC, which might negatively affect the trajectory of cancer, when contributing to related comorbid conditions and, therefore, lead to  poor quality of life. More research is needed. |
| Ibrahim et al. (2021)(4) | United States | Breast Cancer Survivors | To provide a longitudinal assessment of depression and cognitive impairment induced by taxane-based chemotherapy in women with breast cancer after 6 months of treatment | MEDLINE and Embase | Longitudinal  studies | (n=11) | Attention and concentration, depression, and executive function domains had significant chemotherapy-induced impairment across all comparison types. |
| Pizzoferrato et al. (2021)(6) | France | ovarian cancer | To describe the prevalence of pelvic floor disorders and sexuality in women with ovarian cancer | Medline | Mostly cross-sectional | (n=18) | About half of the women are sexually active after surgical treatment with high reported rates of dyspareunia (40–80%) and vaginal dryness (60–80%). Compared with healthy women, some authors found that OC patients had greater problems with loss of desire and poorer sexual function scores. |
| Ebell et al. (2015)(7) | Georgia | Ovarian cancer | To determine the accuracy of individual symptoms and combinations of symptoms for the diagnosis of ovarian cancer. | MEDLINE | Case-control and cohort studies | (n=17) | The highest positive likelihood ratios (LRsþ) were found for abdominal or pelvic pain (LRþ, 10.4); abdominal or pelvic bloating (LRþ, 9.3); loss of appetite presence of abdominal mass (LRþ, 30.0); abdominal distension or increased girth (LRþ, 16.0); (LRþ, 9.2); and a family history of ovarian cancer (LRþ, 7.5). |

1. Watts S, Prescott P, Mason J, McLeod N, Lewith G. Depression and anxiety in ovarian cancer: a systematic review and meta-analysis of prevalence rates. BMJ Open [Internet]. 2015/12/02. 2015;5(11):e007618. Available from: symptom

2. Alanazi MT, Alanazi NT, Alfadeel MA, Bugis BA. Sleep deprivation and quality of life among uterine cancer survivors: systematic review. Support Care Cancer [Internet]. 2022;30(3):2891–900. Available from: https://doi.org/10.1007/s00520-021-06589-9

3. Palagini L, Miniati M, Massa L, Folesani F, Marazziti D, Grassi L, et al. Insomnia and circadian sleep disorders in ovarian cancer: Evaluation and management of underestimated modifiable factors potentially contributing to morbidity. J Sleep Res. 2022;31(3):1–13.

4. Ibrahim EY, Domenicano I, Nyhan K, Elfil M, Mougalian SS, Cartmel B, et al. Cognitive Effects and Depression Associated With Taxane-Based Chemotherapy in Breast Cancer Survivors: A Meta-Analysis. Front Oncol. 2021;11(April).

5. Roussin M, Lowe J, Hamilton A, Martin L. Factors of sexual quality of life in gynaecological cancers: a systematic literature review. Arch Gynecol Obstet [Internet]. 2021;304(3):791–805. Available from: https://doi.org/10.1007/s00404-021-06056-0

6. Pizzoferrato AC, Klein M, Fauvet R, Durand C, Foucher F, Sardain H, et al. Pelvic floor disorders and sexuality in women with ovarian cancer: A systematic review. Gynecol Oncol. 2021;161(1):264–74.

7. Ebell MH, Culp MB, Radke TJ. A Systematic Review of Symptoms for the Diagnosis of Ovarian Cancer. Am J Prev Med [Internet]. 2016;50(3):384–94. Available from: http://dx.doi.org/10.1016/j.amepre.2015.09.023
