# Supplementary material for: A multi-method approach to selecting PRO-CTCAE symptoms for patient-reported outcome in women with endometrial or ovarian cancer undergoing chemotherapy
Source: J Patient Rep Outcomes. 2023 Jul 18;7:72. doi: 10.1186/s41687-023-00611-w (PMC10354345; doi:10.1186/s41687-023-00611-w)
Supplement: Supplementary file 4 — Additional file 4. Comparison of symptoms in various questionnaires and outcome sets. [file 41687_2023_611_MOESM4_ESM.docx]

| MedDRA system organ classes [1] | Core outcome set identified by Reeve et al.[^2^] and Donovan et al.[3] | Symptoms included in MOST T24 (MOST v2)[4][5] | Symptoms included in Webster et al. [6] | Symptoms included in the present tool |
| --- | --- | --- | --- | --- |
| Gastrointestinal disorders |  |  |  |  |
|  | Constipation^2^ | Constipation | Constipation | Constipation |
|  | Diarrhea^2^ | Diarrhea | Diarrhea | Diarrhea |
|  | Nausea^2^ | Nausea | Nausea | Nausea |
|  | Bloating ^3^ | Abdominal pain, discomfort, and/or cramps | Bloating | Bloating |
|  |  | Abdominal swelling, bloating and/or fullness |  |  |
|  | Vomiting^3^ | Vomiting | Vomiting |  |
|  | Cramping^3^ |  |  |  |
|  | Indigestion^3^ | Indigestion |  |  |
|  |  | Sore mouth or throat |  | Mouth/throat sores |
|  |  | Difficulty swallowing |  |  |
| General disorders and administrative site conditions |  |  |  |  |
|  | Fatigue^2^ | Fatigue (tiredness) | Fatigue | Fatigue |
| Metabolism and nutrition disorders |  |  |  |  |
|  | Anorexia (appetite loss)^2^ | Loss of appetite | Decreased appetite | Decreased appetite |
|  | Weight gain^3^ |  |  |  |
|  | Weight loss^3^ |  |  |  |
|  |  | Poor appetite (or feeling full quickly) |  |  |
|  |  | Trouble eating |  |  |
| Musculoskeletal and connective tissue disorders |  |  |  |  |
|  | Pain ^2^ | Pain (all and anywhere) | General pain |  |
|  | Abdominal pain^3^ | Abdominal pain, discomfort, and/or cramps | Abdominal pain | Abdominal pain |
|  |  |  | Joint pain | Joint pain |
|  |  |  |  | Muscle pain |
| Nervous system disorders |  |  |  |  |
|  | Sensory neuropathy^2^ | Numbness or pins and needles | Numbness & tingling | Numbness & tingling |
| RENAL AND URINARY DISORDERS |  |  |  |  |
|  |  | Bladder problems |  |  |
| Reproductive system and breast disorders |  |  |  |  |
|  | Sexual dysfunction^3^ |  | Decreased libido | Decreased libido |
|  |  |  | Pain with sexual intercourse | Vaginal dryness |
| Respiratory, thoracic, and mediastinal disorders |  |  |  |  |
|  | Dyspnea^2^ | Shortness of breath | Shortness of breath | Shortness of breath |
| Psychiatric disorders |  |  |  |  |
|  | Anxiety (includes worry)^2^ | Anxiety (feeling worried) | Anxious | Anxious |
|  | Cognitive problems^2^ | Trouble concentrating | Concentration | Concentration |
|  |  |  | memory | Memory |
|  | Depression (includes sadness)^2^ | Depression (feeling sad) | Discouraged | Discouraged |
|  |  |  | Sad | Sad |
|  | Fear of recurrence/disease progression^2^ |  |  |  |
|  | Insomnia^2^ | Trouble sleeping | Insomnia | Insomnia |
|  |  | Physical well-being |  |  |
|  |  | Emotional well-being |  |  |
|  |  | Overall well-being |  |  |
| Skin and subcutaneous tissue disorders |  |  |  |  |
|  |  | Hair loss | Hair loss |  |
|  |  | Skin rash | Rash |  |
|  |  | Sore hands and feet |  |  |
| Investigations |  |  |  |  |
|  |  | Altered sense of taste | Taste changes |  |
|  |  |  | Swelling |  |
| Unique symptoms, total | **21** | **24** | **24** | **21** |
| Similar to our PRO tool | **71%** | **52%** | **86%** |  |
| Recall period | **3–4 weeks** | **7 days** | **7 days** |  |

*MedDRA* Medical Dictionary for Regulatory Activities, *MOST* Measure of Ovarian cancer Symptoms and Treatment concerns – MOST-T24 (MOST v2), PRO Patient-Reported Outcomes

1. Medical Dictionary for Regulatory Activities (MEDDRA). https://www.meddra.org/. Accessed 10 Nov 2022

2. Reeve BB, Mitchell SA, Dueck AC, et al (2014) Recommended patient-reported core set of symptoms to measure in adult cancer treatment trials. J Natl Cancer Inst 106:. https://doi.org/10.1093/jnci/dju129

3. Donovan KA, Donovan HS, Cella D, et al (2014) Recommended patient-reported core set of symptoms and quality-of-life domains to measure in ovarian cancer treatment trials. J Natl Cancer Inst 106:10–13. https://doi.org/10.1093/jnci/dju128

4. GCIG GYNECOLOGIC CANCER INTERGROUP. https://gcigtrials.org/content/most. Accessed 23 Nov 2022

5. King MT, Stockler MR, O’Connell RL, et al (2018) Measuring what matters MOST: validation of the Measure of Ovarian Symptoms and Treatment, a patient-reported outcome measure of symptom burden and impact of chemotherapy in recurrent ovarian cancer. Qual Life Res 27:59–74. https://doi.org/10.1007/s11136-017-1729-8

6. Webster EM, Burke WM, Ware HM, et al (2018) Patient reported outcomes in evaluation of chemotherapy toxicity in women with gynecologic malignancies: A pilot study. Gynecol Oncol 150:487–493. https://doi.org/10.1016/j.ygyno.2018.07.008
